# The Antioxidant Activities of Ethanolic, Methanolic, Ethyl Acetate, and Aqueous Extracts of the Endemic Species, Lavandula *mairei* Humbert (A Comparative Study between Cold and Hot Extraction)

**DOI:** 10.4314/ejhs.v32i6.21

**Published:** 2022-11

**Authors:** Ridwane Ghanimi, Ahmed Ouhammou, Yassine El Atki, Mohamed El Hassan Bouchari, Mohamed Cherkaoui

**Affiliations:** 1 Laboratory of Pharmacology, Neurobiology, Anthropobiology, Environment, and Behaviour, Department of Biology, Faculty of Sciences Semlalia, Cadi Ayyad University, Marrakech, Morocco; 2 Laboratory of Microbial Biotechnologies, Agrosciences and Environment (BioMAgE), Agrosciences, Phytobiodiversity, and Environment Team, Regional Herbarium ‘MARK’, Department of Biology, Faculty of Sciences Semlalia, Cadi Ayyad University, Marrakech, Morocco; 3 Laboratory of Physiology Pharmacology and Environmental Health, Department of Biology, Faculty of Sciences Dhar Mehraz, Sidi Mohamed Ben Abdellah University, Fez, Morocco; 4 Departement of Life Sciences, Faculty of Technical Sciences, Sultan Moulay Slimane, BéniMellal, Morocco

**Keywords:** Antioxidant activity, Lavandula mairei, Extracts, Maceration, Soxhlet

## Abstract

**Background:**

Medicinal plants have been used for therapeutic purposes and have shown important biological properties. This study aimed to evaluate for the first time the antioxidant activities, total flavonoid, and total phenolic contents of Lavandula mairei Humbert. The ethanol, methanol, ethyl-acetate, and water extracts were used for this purpose.

**Methods:**

The antioxidant activities were assessed in vitro by free radical scavenging activity against 2,2-diphenyl-1-picrylhydrzyl (DPPH), ferric reducing antioxidant power (FRAP), and total antioxidant capacity (TAC). The total flavonoid and phenolic contents were determined spectrophotometrically with gallic acid and Quercetin as standards.

**Results:**

In either Soxhlet or maceration methods, the flavonoids and the total phenolic contents were significantly higher in the methanolic extract (P<0.05) compared to other extracts. The total flavonoid content of L. mairei ranged between 119 and 224.6 mg QE/g DW for Soxhlet extracts and from 111.8 to 148.51 mg QE/g DW for maceration extracts. While the total phenolic content was between 35.12 and 99.37 mg GAE/g DW for Soxhlet extracts and 27.63 to 58.99 mg GAE/g DW for maceration extracts. In either the Soxhlet or maceration method, the highest total antioxidant capacity (TAC) was obtained using the ethanolic extract, while the aqueous extract had the highest antioxidant activity for DPPH and FRAP assays.

**Conclusion:**

These results showed that Lavandula mairei Humbert has great potential to be a promising candidate for natural plant sources of antioxidants.

## Introduction

Reactive oxygen species (ROS) such as superoxide anion, hydroxyl radicals, and hydrogen peroxide are free radicals implicated in several diseases (1). In contrast, the human body has an endogenous antioxidant system that maintains the intracellular redox potential (2). However, endogenous antioxidants are not sufficient in case of severe or continuous oxidative stress (3). Therefore, the continuous supply of exogenous antioxidants is essential to maintain an adequate level of antioxidants (4).

Medicinal plants are rich in secondary metabolites that are known to be protective against many diseases such as cardiac disorder and cancer (5–8). These secondary metabolites are generally phenolic acids, flavonoids, tannins, stilbenes, and lignans (9). As a result, there is a growing demand for natural products with a high content of phenolic compounds because of their biological characteristics, including free radical scavenging (10). Moreover, antioxidants are used in the food industry to extend shelf life (11). However, it is widely accepted that synthetic antioxidants such as butylated hydroxyanisole (BHA) and butylated hydroxytoluene (BHT) should be substituted with natural antioxidants due to their potential risk to health and their toxicity (12). For these reasons, the study of antioxidant capacities and the discovery of new sources containing phenolic compounds are crucial (13).

The present study aimed to investigate, for the first time, the total phenolic, the flavonoids contents, and the antioxidant activities of Lavandula mairei Humbert using four extracts (Water, Ethyl Acetate, ethanol, and methanol) and two extraction methods (maceration and soxhlet).

## Methods

Plant materials: Aerial parts of Lavandula mairei Humbert were collected in Mai 2019 from the Al-Haouz region (Morocco). The plant material was identified by one of the co-authors in the regional herbarium “MARK” of the FSSM-Marrakech, University Cadi Ayyad (Morocco).

**Preparation of the extracts**: The samples (10g) were dried in the shade at room temperature and then powdered. The extraction was performed with four solvents (ethanol, methanol, ethyl acetate, and water) and two extraction technics (Soxhlet and maceration). The solid to liquid ratio was 1/20 using a Soxhlet extractor for 8 h, while the maceration result was filtered and then concentrated to dryness under vacuum at 40°C. The extracts were then stored at 4°C in a refrigerator.

**Total phenolic contents**: The total phenolic content of the extracts was measured according to the Folin-Ciocalteu method (14). The absorbance was read at 760 nm after 2 h of reaction in the dark at room temperature. Regarding the construction of the calibration curve, Gallic acid was used as the standard. The total phenol contents were expressed as milligrams of Gallic acid equivalents per gram of dry weight (mg GAE/g DW).

**Total flavonoid contents**: The total flavonoid contents of the extracts were measured by the aluminum chloride colorimetric assay (15). In a 10 ml volumetric flask, 1 ml of each sample was added to 4 ml of distilled water then 0.30 ml of 5% NaNO2 was added. Five minutes later 0.3 ml of 10 % AlCl3 was added to react for 6 min. After that, 2 ml of NaOH (1M) was added and the total was made up to 10 ml with distilled water. The solution was mixed and absorbance was measured against the blank at 510 nm. Quercetin was used as a standard and the total flavonoid contents were expressed as mg Quercetin equivalents per gram of dry weight (mg QE/g DW).

**Antioxidant activity by DPPH (radical scavenging activity)**: The capacity of the extract to scavenge the DPPH radical was measured using the method described by Wu et al. (2003). A volume of 100 µl of various concentrations of the extracts or standard was added to 1.4 ml of the ethanolic solution containing 0.1 mM of DPPH (2, 2-diphenyl-1 picrylhydrazyl). The mixture absorbance was measured at 517 nm by a spectrophotometer after 30 min of incubation in the dark at room temperature. The percentage of inhibition was expressed as I% = ((A0-As)/A0)*100. Where A0 is the Blank absorbance and As is the sample absorbance. Butylated hydroxytoluene (BHT) was used as a positive control and the IC50 values were calculated as the concentration causing 50% inhibition of the DPPH.

**Antioxidant activity by Ferric Reducing Antioxidant Power (FRAP) Assay**: The reducing power activity of the extracts was measured using the method of Oyaizu 1986 (17). A set of 500 µl of phosphate buffer (0.2 M, pH 6.6) and 500 µl of potassium ferricyanide [K3Fe(CN)6] 1% were mixed with 200 µl of the extracts. The mixture was incubated in a bain-marie for 20 min at 50°C. A volume of 500 µl of trichloracetic acid (TCA) 10% was used to acidify the mixture. The obtained solution was then centrifuged for 10 min at 3000 rpm. 500 µl of the solution's upper layer was mixed with 100 µl of FeCl3 (0.1%) and 500 µl of distilled water. The absorbance was measured at 700 nm and Quercetin was used as a standard. EC50 (mg/ml) was calculated by plotting the absorbance against the corresponding concentration (EC50 concentration corresponding to 0.5 of absorbance).

**Antioxidant activity by total antioxidant capacity**: The analysis was based on the reduction of Mo (VI) to Mo (V) and the resulting formation of a green phosphate/Mo(V) complex at acidic pH (18). A volume of 25 µl of extraction was added to 1 ml of reagent solution (sulfuric acid 0.6 mol/L, sodium phosphate 28 mmol/L, and ammonium molybdate 4mmol/L). The mixtures were incubated in a bain-marie for 90 min at 95° C and then cooled to ambient temperature. The absorbance was measured at 695 nm, and the total antioxidant activity was given as an ascorbic acid equivalence number (mg AAE/g DW).

**Statistical analysis**: All tests were performed in triplicate, and the mean values and standard deviations were calculated by using SPSS 20. The results were compared by one-way ANOVA followed by Tukey-test, using the same software. Differences were considered to be significant at P < 0.05.

## Results

**Figure 1:**
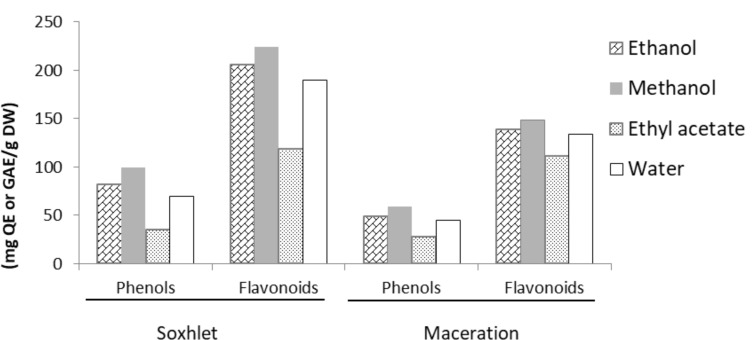
Total phenolic and flavonoid contents of different extracts from L. mairei by Soxhlet and maceration methods. The total flavonoid content expressed as mg QE/g DW and the total phenolic content expressed as mg GAE/g DW

Total flavonoid and phenolic contents: As shown in table 1, the total phenolic and flavonoid contents were determined in comparison with Gallic acid and Quercetin standards. The results were expressed as gallic acid and Quercetin equivalents (mg) per gram of dry weight for total phenolic and flavonoid contents, respectively. The total flavonoid content of L. mairei ranged between 119 and 224.6 mg QE/g DW for Soxhlet extracts and from 111.8 to 148.51 mg QE/g DW for maceration extracts. While the total phenolic content was between 35.12 and 99.37 mg GAE/g DW for Soxhlet extracts and 27.63 to 58.99 mg GAE/g DW for maceration extracts. Furthermore, the methanolic extract contained significantly a higher concentration of flavonoids and phenols for the two extraction methods (Figure 1) than the other tested extracts.

**Table 1:** Total phenolic and flavonoid contents of different extracts from L. mairei by Soxhlet and maceration methods

	Soxhlet		Maceration	
	Phenols (mg GAE/g DW)	Flavonoids (mg QE/g DW)	Phenols (mg GAE/g DW)	Flavonoids (mg QE/g DW)
**Ethanol**	82.44±2.67A	206.3±4.65A	48.69±1.59A	139.2±2A
**Methanol**	99.37±1.78B	224.6±3.06B	58.99±1.6B	148.51±2B
**Ethyl acetate**	35.12±1.34C	119±2.76C	27.63±1.09C	111.8±2.2C
**Water**	69.76±1.01D	190.2±2.78D	45.33±1.45A	134.3±2.6A

Data are expressed as mean ± standard deviation (n=3). In each column, different letters are significantly different by the Tukey test (P<0.05).

**Antioxidant activities (DPPH, FRAP, and TAC)**: The results in Table 2 represent the antioxidant activity by DPPH, FRAP, and total antioxidant activity (TAC) of the four extracts (Ethanol, Methanol, Ethyl acetate, and Water) during hot extraction (Soxhlet) and cold extraction (Maceration).

**Table 2:** Antioxidant activities of different extracts from L. mairei by Soxhlet and maceration methods Soxhlet Maceration

	Soxhlet			Maceration		

	DPPH (mg/ml)	FRAP (mg/ml)	TAC (mg AAE/g DW)	DPPH (mg/ml)	FRAP (mg/ml)	TAC (mg AAE/g DW)
**Ethanol**	0.73±0.014^A^	0.51±0.019^A^	176.34±2.4^A^	0.648±0.085^A^	0.973±0.009^A^	123.45±5.3^A^
**Methanol**	0.41±0.009^B^	0.31±0.033^B^	161.53±4.9^B^	0.355±0.005^B^	0.814±0.013^B^	120.84±2.7^A^
**Ethyl acetate**	1.126±0.005^C^	0.786±0.071^C^	97.59±3.81^C^	1.086±0.003^C^	1.396±0.039^C^	80.07±5.36^B^
**Water**	0.359±0.001^D^	0.054±0.002^D^	135.55±3.3^D^	0.409±0.002^B^	0.205±0.002^D^	102.68±5.1^C^

Data are expressed as mean ± standard deviation (n=3). In each column, different letters are significantly different by the Tukey-test (P<0.05)

Concerning Soxhlet extraction, the highest total antioxidant capacity (TAC) was obtained using the ethanolic extract (176.34±2.4 mg AAE/g DW), while the aqueous extract had the highest antioxidant activity for DPPH (0.359±0.001 mg/ml) and the FRAP (0.054±0.002 mg/ml) methods. The four investigated extracts exhibited significant differences at (P<0.05) among free radical scavenging DPPH, ferric reducing capacity (FRAP), and total antioxidant activity (TAC) assays.

maceration extraction, ethanolic and methanolic extracts presented the highest total antioxidant capacity (123.45±5.3 and 120.84±2.7 mg AAE/g DW, respectively). On the other hand, the best activity for the FRAP assay was found in the aqueous extract (0.205±0.002 mg/ml). While methanolic (0.355±0.005 mg/ml) and aqueous (0.409±0.002 mg/ml) extracts showed the best antioxidant capacity for DPPH free radical scavenging assay.

## Discussion

In our study as in the study conducted by El Atki et al. (2019), the methanolic extract has shown higher flavonoid contents compared to other extracts. Therefore, methanol is the best solvent to extract flavonoids and phenolic compounds from medicinal plants (20). Many studies revealed the strong antioxidant activity of phenolic and flavonoid compounds (21,22) and other therapeutic properties including anticancer (23), anti-inflammatory (24), antiviral (25), and antibacterial capacities (26). Moreover, various studies assessed the antioxidant activities and revealed significant differences between ethanol, methanol, ethyl acetate, and water extracts (27–30).

In conclusion, the present study is the first to evaluate the antioxidant capacity of different extracts of Lavandula mairei Humbert. The flavonoids and the total phenolic contents were significantly higher in the methanolic extract (P<0.05) compared to other extracts (in either Soxhlet or maceration methods). The high contents of phenols and flavonoids indicated that these compounds could contribute to the antioxidant capacity of this plant. The Lavandula mairei Humbert has the potential to be a promising candidate for natural plant sources of high-value antioxidants.
